# Parkinson’s disease population-wide registries in the United States: Current and future opportunities

**DOI:** 10.3389/fdgth.2023.1149154

**Published:** 2023-03-22

**Authors:** Allan D. Wu, Andrew M. Wilson

**Affiliations:** ^1^ Division of Movement Disorders, Department of Neurology, Feinberg School of Medicine, Northwestern University, Chicago, IL, United States; ^2^ Department of Neurology, David Geffen School of Medicine, University of California Los Angeles (UCLA), Los Angeles, CA, United States; ^3^ Stanley Manne Children’s Research Institute, Ann & Robert H. Lurie Children’s Hospital, Chicago, IL, United States; ^4^ Department of Neurology, Greater Los Angeles VA, Los Angeles, CA, United States

**Keywords:** Parkinson's disease, parkinsonism, registries, epidemiology, population informatics

## Abstract

Parkinson’s disease (PD) is a neurodegenerative disease with both genetic and environmental risk factors. Efforts to understand the growing incidence and prevalence of PD have led to several state PD registry initiatives in the United States. The California PD Registry (CPDR) is the largest state-wide PD registry and requires electronic reporting of all eligible cases by all medical providers. We borrow from our experience with the CPDR to highlight 4 gaps to population-based PD registries. Specifically we address (1) who should be included in PD registries; (2) what data should be collected in PD case reports; (3) how to ensure the validity of case reports; and (4) how can state PD registries exchange and aggregate information. We propose a set of recommendations that addresses these and other gaps toward achieving a promise of a practical, interoperable, and scalable PD registry in the U.S., which can serve as a key health information resource to support epidemiology, health equity, quality improvement, and research.

## Introduction

Parkinson’s disease (PD) is the most rapidly growing neurodegenerative disease across the globe (1). Epidemiology studies, using claims datasets, have estimated prevalence and incidence of PD (2, 3), and observational cohort studies have identified both environmental and genetic risk factors for the development of PD (4–6). To expand upon this work, true population-wide PD registries, leveraging real-time electronic health record (EHR) data associated with clinical care, hold promise to address more comprehensive questions about epidemiological risk factors, treatment, healthcare utilization, and outcomes across the wide diversity of people and community settings.

In the United States, statewide PD surveillance registries are growing in momentum to assess the prevalence, incidence, and distribution of cases and to support public health education, outreach, and research. Nebraska was the first statewide PD registry (1997), requiring reporting of new PD cases (7). The California Parkinson’s Disease Registry (CPDR) was established in 2005 to determine the incidence and prevalence of PD in California, to examine disparities in PD risk, and to conduct demographic and epidemiological research. The CPDR started requiring mandatory reporting of all PD cases in 2018 (8, 9). Multiple states have smaller registries or legislation pending for PD or neurodegenerative registries (10, 11). In parallel, Congress authorized the Centers for Disease Prevention and Control (CDC) to develop a National Neurologic Conditions Surveillance System (NNCSS), with initial conditions being PD and Multiple Sclerosis (12). To date, efforts to align state PD registries to form an effective network of statewide PD registries are limited.

In this *Perspective*, we discuss the CPDR as an all-electronic, near real-time PD registry and the largest current example of a state PD registry. The CPDR requires all providers to report encounters where PD is treated or diagnosed, regardless of encounter type or specialty—a particularly broad set of criteria. Cases can be reported in real-time using electronic health record (EHR) case reports (or near real-time in quarterly batches); an online portal is used for manual reporting of individual cases. As of 2021, the CPDR has received 534,583 reports from 550 reporting entities across most counties, covering 93,928 unique PD patients (13). Reporting from California practices is not yet considered complete and no prevalence estimates have been released. For researchers, a data disclosure policy and procedure was released in 2021.

The CPDR also exemplifies many challenges and gaps faced by population-wide PD registry design, implementation, and usability. To help address these gaps, the Michael J. Fox Foundation for Parkinson’s Research recently supported an independent project at the University of California, Los Angeles (the UCLA-CPDR-EHR PD UCE-PD project). The UCE-PD project aims were to assess the accuracy and completeness of data collected by automated means at a single large academic site and to develop, implement, and demonstrate a framework of tools to improve upon CPDR accuracy and completeness. The UCE-PD project was led by a multidisciplinary team including movement disorders specialists, general neurologists, and primary care physicians with expertise representing clinical practice, epidemiology, clinical informatics, and health services research.

We borrow from our experience with the CPDR and UCE-PD project to highlight 4 gaps in population-based PD registries. Specifically we address (1) who should be included in PD registries; (2) what data should be collected in case reports; (3) how to ensure the validity of case reports; and (4) how can PD registries exchange information? We conclude by presenting a list of recommendations to consider as next steps toward realizing a population-wide PD registry.

## Gap 1: who should be included in a PD registry?

The clinical diagnosis of PD can be challenging as there is no confirmatory test or biomarker. Current diagnostic criteria for PD rely on clinical expertise and factors that are uncommonly coded reliably or accurately in EHRs (14, 15). There are circumstances when the diagnosis of PD cannot be made with confidence (16), particularly early cases of parkinsonism or those confounded by alternate causes (e.g., drug-related or vascular). The diagnosis of PD is also confounded by related, though distinct, neurodegenerative parkinsonism syndromes (NPS) such as progressive supranuclear palsy (PSP), corticobasal syndrome (CBS), multiple systems atrophy (MSA), or dementia with Lewy bodies (DLB), which may only become clinically clear after years of being diagnosed with PD. Notably, persons with NPS are of interest to PD registries because they may share epidemiological risks and have similar health resource needs as persons with PD (PwPD). Excluding NPS risks reducing potential value in a PD registry.

There is also no consensus EHR phenotype for PD. A combination of diagnosis codes, medications, provider specialties, and lookback intervals have been used by many published algorithms for detecting PD from EHR data and support the ability to detect PD or NPS (17–21). Unfortunately, estimates are that only 75%–82% of cases of PD detectable by codes are actually PD (21–23). Such issues, well-known among neurologists, researchers, and clinicians, contribute to some skepticism for cases included (or not included) in registries. Additionally, performance of algorithms are challenging to interpret because of variation in whether the focus is on the detection of PD itself, PD with NPS, or parkinsonism in general (17). Further, algorithms developed in one system have rarely been tested using data across differing systems, and consensus algorithms have not yet emerged (24).

**Figure 1:**
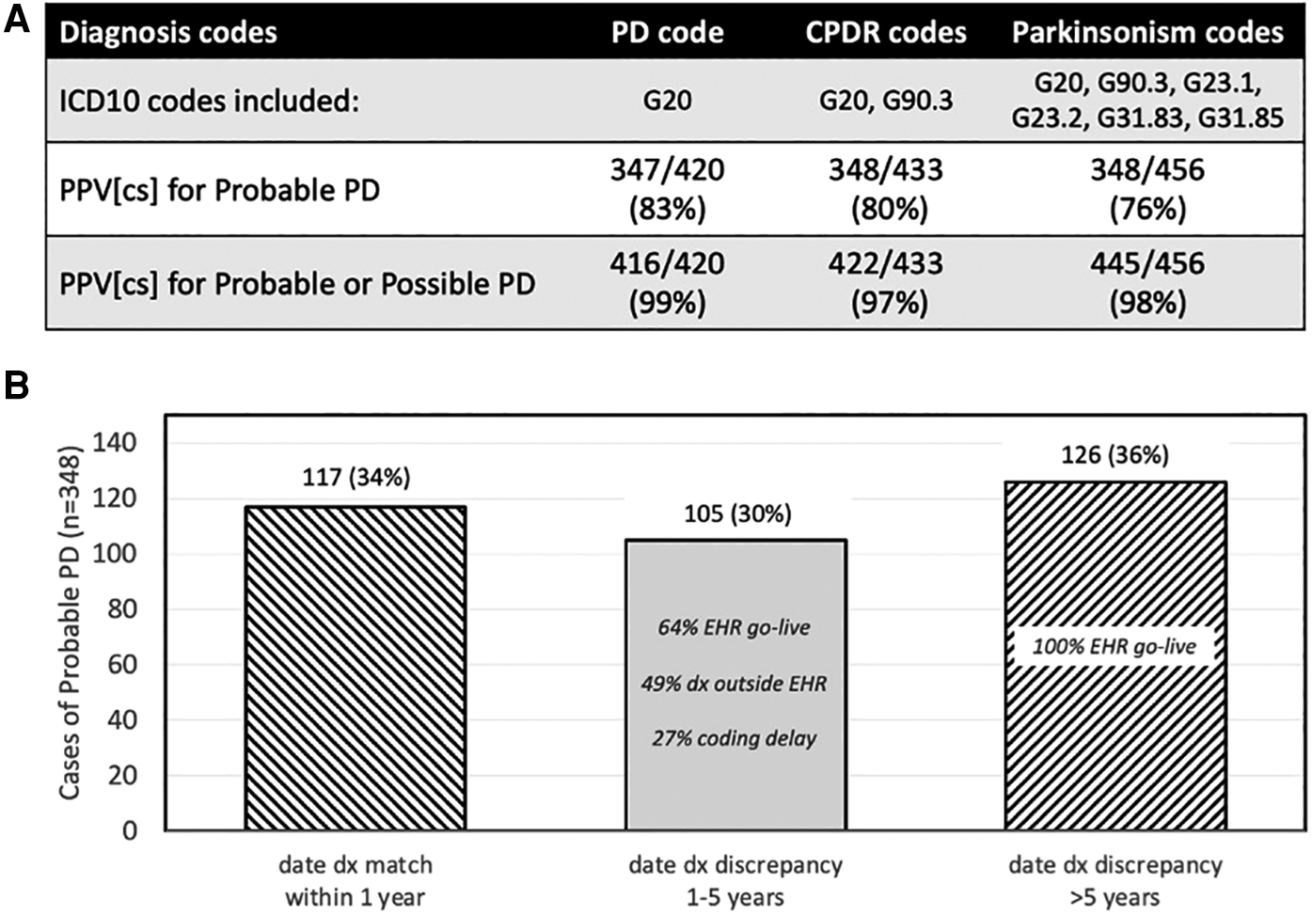
Performance of CPDR diagnosis codes and date of diagnosis. (**A**) Effect of different ICD10 trigger codes on case identification of Probable or Possible PD in a cross-sectional (CS) sample of cases. Probable and Possible PD assignment was made by a neurologist after manual chart review. The lowest positive predictive value (PPV) for Probable or Possible PD was with CPDR codes. (**B**) For cases of Probable PD (*n* = 348), the date of diagnosis discrepancy between the CPDR reported date and the date from chart review was within 1 year in 34% of cases. The percentage of cases with discrepancies less than or more than 5 years (middle and right bar, respectively), and the reasons for those discrepancies, are shown. Some cases had more than one discrepancy reason. EHR go-live 5 years prior to data collection resulted in a floor for automated CPDR-reported dates.

The CPDR relies only on ICD10 diagnosis codes (G20 and G90.3) to trigger encounters to report. While G90.3 is intended to identify MSA, other NPS were excluded. The G20 code represents PD, but is also used when the clinician codes for less certain parkinsonism “not otherwise specified.” When the UCE-PD team reviewed a sample of 456 patients identified using six parkinsonism codes, we found that the two code CPDR combination had a lower positive predictive value for PD than G20 alone or the broader set of parkinsonism codes (Figure 1A).

## Gap 2: what data should be collected in PD data reports?

Currently, individual statewide PD registries separately develop data specifications, which limits harmonization of collected data and reduces the potential benefit of such registries. Consensus standards for data elements recommended for population-wide PD registries have yet to be established.

The CPDR experience is illustrative of the challenge of determining a minimum required specification across a diverse healthcare system. Initial CPDR proposed specifications included both administrative data (reporting entity, patient demographics, provider information) and required available clinical data elements, such as PD symptoms, medications and comorbidities (25). Due to non-standard and variable nature of how clinical data elements are documented among different hospital and clinic settings, advocacy groups responded with concerns about reporting feasibility and burden. The final CPDR specification places nearly all clinical data elements into an optional category, with exceptions being encounter diagnosis codes and the date of diagnosis [(9), Supplementary Material S1]. This oversimplification of required PD data elements limited clinical utility and reduced some ability to de-duplicate or validate cases reported.

Some data elements that may be considered for PD registries may not be easily, reliably, or accurately captured. For example, the CPDR specification requires that each report include a date of diagnosis. Even mature cancer registries, where this date is a key data element and anchored by pathological confirmation, struggle to obtain this information from oncology specialty practices (26). To ensure success of initial CPDR implementation, the registry allows the earliest date of a trigger diagnosis on the Problem List or the earliest encounter date to be used as the date of diagnosis (9). This data definition favors completeness of data reported with potential risk to accuracy. When reviewed in a sample of 348 Probable PD cases, the UCE-PD team found that the date of diagnosis reported by CPDR specifications was accurate within 1 year in 34% of cases when compared to that by gold-standard manual chart review (Figure 1B). This work emphasizes that certain data elements being considered as specifications for a PD registry should be assessed for quantifiable risk of accuracy or completeness.

## Gap 3: how should PD case reports and registry data be validated?

Quality assurance (QA) and case validation ensure that registries are capturing data that are complete and high quality. QA is arguably even more important in real-world, EHR-driven, population-wide registries, where data are created as a byproduct of clinical care (27). Because PD and NPS do not have a definitive biomarker or standard nomenclature (unlike cancer staging), the need for a transparent and robust validation process to build confidence among stakeholders is important to include in PD registry design.

Cancer registries, consolidated under the CDC National Program of Cancer Registries (NPCR), cover 97% of the US population and collect timely data on incidence, treatments, and outcomes (28, 29). To achieve this, resources are available to train and certify cancer registry abstractors, usually near the point of care. As such, population cancer registries focus abstractor efforts on the collection and submission of high quality data at the source. Many existing Parkinson disease registry efforts similarly collect high quality data, requiring considerable resources, from selected movement disorder specialty sites (30).

This point of care approach is not practical for population-scale PD registries where cases are reported across a wide spectrum of medical practices. Validation within large registries typically sample cases and compare them against gold-standard neurologic assessment or manual chart review. The outcome is to assess whether variations in PD data in the registry between sites are due to differences in coding practices (17), distribution of care (3), or represent actual differences in incidence or prevalence. The CPDR provides completeness data to reporting sites, but has not yet adopted guidance for validating cases reported to the registry.

For the UCE-PD project, we developed a proof of principle validation workflow for CPDR-eligible cases. The strategy used was to select cases for review, have trained abstractors manually review charts using a standardized abstraction tool, and have experienced adjudicators confirm the PD diagnosis classification by reviewing summary information from the abstraction tool. QA tools were developed including abstractor training modules, feedback sessions, and inter-rater dual abstraction reliability checks. Challenging cases were escalated for further chart review to neurologists to finalize an adjudicated classification. Each final case classification would represent the gold-standard for validation purposes.

This validation process is theoretically scalable because of its potential federated approach. The validation abstraction process and associated QA checks can be conducted within each local site. Importantly, clinician review would not be required for most adjudications. Applied across all sites reporting PD cases, this process can provide standardized validation information that can help enhance the trust of patients, clinicians, and researchers participating in PD registries.

## Gap 4: how can PD registries exchange and aggregate information?

It is unrealistic to think that any one singular registry can house the requisite information to address the epidemiological, clinical, and health services questions of the future. A successful population-wide PD registry will require an interoperability infrastructure that supports data exchange among registries.

Interoperability requires a common standard of codes that represent data elements captured from all EHRs used. To the extent possible, registries will specify mappings of required data elements to standard terminology code sets, such as ICD10 for diagnoses, RxNorm for medications, CPT for procedures, etc. (31). However, some concepts important for PD registries may not yet have a standard code. As an example, movement disorders, as a neurological subspecialty, is not represented in standard specialty taxonomy code sets (32). In circumstances where gaps exist in standard code sets, an interim step can be to partner with health information exchanges (HIE) that could support non-standard data elements of importance.

To illustrate, the UCE-PD team developed a focused data dictionary of symptoms that are commonly encountered in PD. We worked with an EHR vendor (Epic Systems, Verona WI) to create common PD registry data elements within the default EHR system. These symptom data elements are now automatically available and semantically interoperable for all customers within the vendor-specific HIE (Supplementary Material S2).

The technical tools and trust framework of sharing PD registry data are an area for innovation and ongoing evolution. Health Level 7 International (HL7) sets widely used standards for the exchange, integration, sharing, and retrieval of electronic health information. An electronic case report (eCR) standard was released by HL7 in 2017 with data elements that represent a consensus “minimum necessary” for public health case reports (33). Prior to 2019, implementation of eCR by entities reporting to the CPDR was low. As eCR was widely promoted during the Covid pandemic (34), there was a significant increase in entities that supported eCR infrastructure. The CPDR saw an increase in eCR format reports from none in 2019 to 67% of reported cases in 2021 (personal communication, CPDR). Unfortunately, piecemeal adoption of eCR formats by individual county and state public health departments, rather than broad adoption, remains a barrier to full interoperability.

## Discussion

We briefly outlined some of the current state and challenges of developing population-wide PD registries. We discussed CPDR as an example of a statewide PD registry implementation, recognizing the growing momentum toward additional statewide registries in the near future. With this context, we propose a set of recommendations that addresses these and other gaps toward achieving a promise of practical, interoperable, and scalable population-wide PD registries. While this *Perspective* focuses on aspects of a U.S. state implementation, how to adapt these recommendations to international sites must also be considered. The state-by-state (e.g., California, Nebraska) approach that characterizes U.S. public health presents challenges may be less prevalent in centralized healthcare systems. Our vision is that, while the initial population-wide registries will first support use cases of public health surveillance, epidemiology, and assessment of health care utilization, the maturation of broad interoperability frameworks will enable development of these PD registries as key population-wide health information resources. For example, when eventually linkable to other patient outcomes, clinical trial data, quality registries, genomics, and biorepository resources, discoveries can be inferred, developed, and applied at scale as public health interventions to advance access and health equity outcomes, quality improvement initiatives, and research efforts.

*Overview recommendations:*

1. Propose a series of symposia or workshops to develop consensus around a core set of infrastructure decisions to support population-wide PD registries. Participation should include subject matter experts, patient advocacy groups, specialty societies, health system informaticists, state public health departments, and CDC NNCSS to develop broad stakeholder engagement.
2. Develop a vision and mission statement about the role of population-wide and state PD registries. This statement should reflect direct goals supporting public health surveillance, health services equity, and epidemiology research as well as longer-term goals to support efforts in public health intervention, quality improvement, and research. Endorsement of FAIR (Findable, Accessible, Interoperable, and Reusable) principles for data management should be encouraged (35).
3. Develop, publish, and maintain a repository of standards and guidelines. As registries are repositories of systematically collected data, a central set of consensus documentation around use cases, implementation strategies, data collection standard operating procedures, and data dictionaries, as further discussed in points below, will be needed.
4. Develop guidelines at state and federal levels to address common issues for large public health registries including, but not limited to patient confidentiality, the balance of public health vs. right to privacy, ownership of data, reusability of data, and return of benefits of the registry to stakeholders.
5. Evaluate, develop, and share models for financial and resource sustainability for individual state PD registries, exploring partnerships with academia, third-party vendors, federal regulatory agencies, or other solutions. To date, uncertainties in state budgets have adversely affected operations in state-funded registries (i.e., CPDR, Nebraska PD Registry). As an exemplar, a combination of federal, state, and private funds have helped sustain cancer registries in the US (36).

*Scientific considerations:*

**Figure 2:**
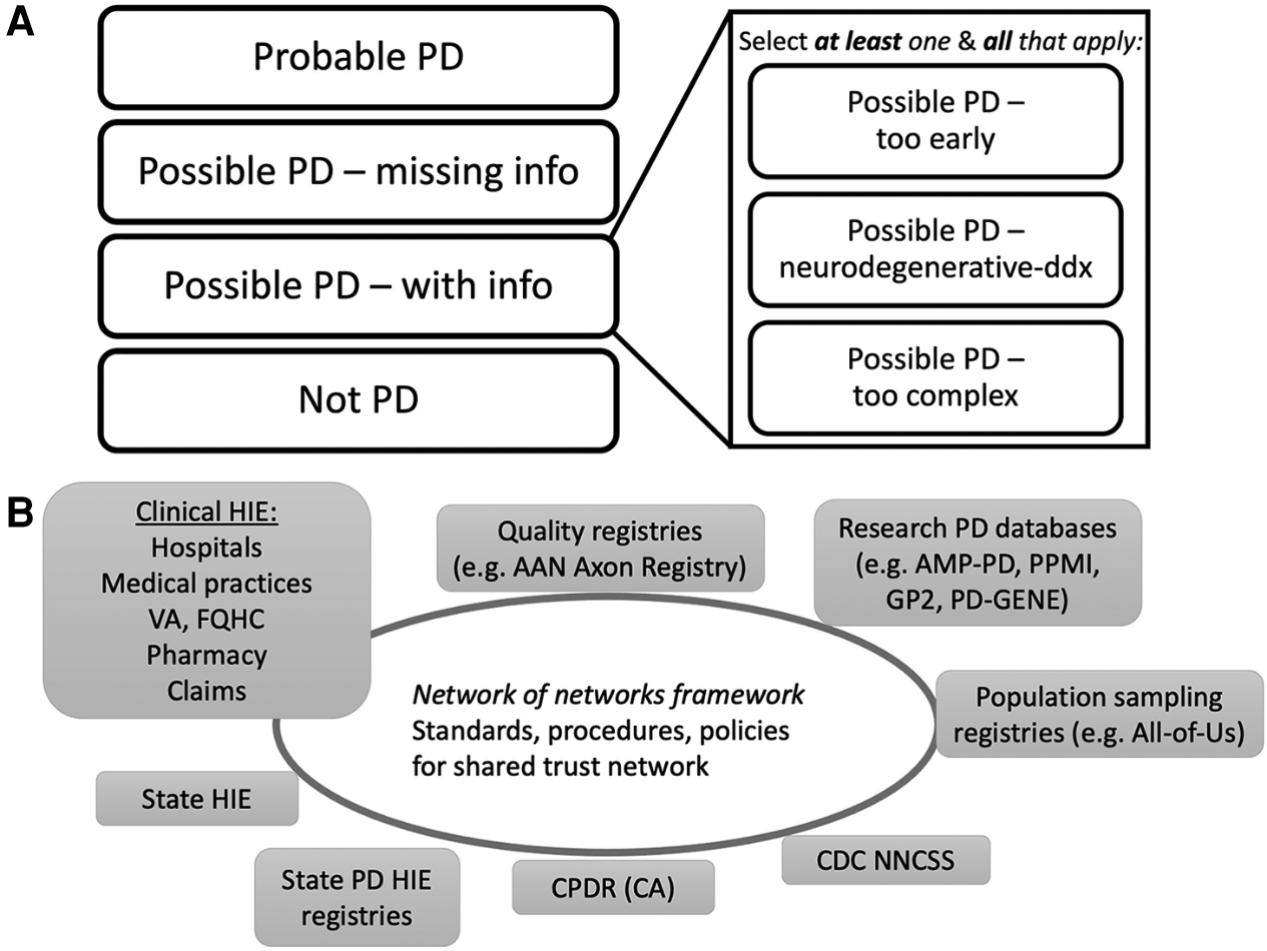
Proposed EHR PD classifications and future interoperability network with PD registries. (**A**) Overview of UCE-PD classification for each reported case at a point in time in PD/parkinsonism registries. Each case has a unique classification (left) with non-exclusive subclassifications for Possible PD (right) (Supplementary Table S2 for details). (**B**) Schematic of a future interoperability network of networks to support electronic health information exchange (HIE) relevant for PD registries. A national trust framework (Trusted Exchange Framework Common Agreement, TEFCA) will facilitate exchanges among PD registries and balance public health mandatory reporting with the sharing of clinical, quality, or research information within appropriate legal, compliance, confidentiality, and privacy policies. Individual state PD registries (CPDR for example) could connect to the framework directly, could form interstate PD-specific HIEs, or connect indirectly *via* other clinical HIEs. Other specific PD registries (quality, research), if part of the trust framework, could request information from population-wide PD registries for relevant context, and vice versa. EHR, electronic health record; VA, Veteran’s Administration; FQHC, federally qualified health center; AAN, American Academy of Neurology; AMP-PD, Accelerating Medicines Partnership Parkinson’s Disease; PPMI, Parkinson’s Progression Markers Initiative; GP2, Global Parkinson’s Genetics Program; PD-GENE, PD GENEration; CDC NNCSS, Centers for Disease Control National Neurologic Conditions Surveillance System; CPDR, California Parkinson’s Disease Registry; CA, California. EHR, electronic health record; VA, Veteran’s Administration; FQHC, federally qualified health center; AAN, American Academy of Neurology; AMP-PD, Accelerating Medicines Partnership Parkinson’s Disease; PPMI, Parkinson’s Progression Markers Initiative; GP2, Global Parkinson’s Genetics Program; PD-GENE, PD GENEration; CDC NNCSS, Centers for Disease Control National Neurologic Conditions Surveillance System; CPDR, California Parkinson’s Disease Registry; CA, California.

6. Encourage inclusive PD/parkinsonism registries that will encompass both PD and NPS. This recommendation is supported by clinical overlap, challenges in detecting early possible PD or NPS, and need to understand scientific, clinical, and health care delivery similarities and differences with both PD and NPS.
  a. Initial CPDR criteria of six ICD10 codes for parkinsonism are a good starting point, but further scientific consensus on reporting criteria is recommended.
7. Support development of a practical intermediate classification system for labelling each case reported in a broadly inclusive PD/parkinsonism registry. The classification should be granular enough to reflect real-world uncertainties in PD diagnosis, yet high-level and discrete enough to facilitate automatic interoperable mapping between state registries.
  a. The UCE-PD team developed a consensus diagnostic classification scheme to account for the variations in data quality and diagnostic uncertainty commonly encountered when validating cases of potential PD. Figure 2A (and Supplementary Material S3) illustrates the UCE-PD consensus nomenclature and conceptual definitions for labelling case reports.
8. Develop a consensus data dictionary of elements recommended for reporting to population-wide PD registries, prioritizing those elements that are readily available (feasible and accurate) and are essential to case de-duplication and validation. Such a data dictionary will ensure a common base of terminology for interoperable data exchange among PD registries.
  a. Elements range from demographic elements (most feasible), administrative data elements (e.g., encounter dates and types, specialties, medications; feasible, some variability), to clinical symptom, diagnostic certainty, and disease severity elements (most challenging to standardize within real-time workflows).
9. Recommend a basic, minimum dataset standard for mandatory reporting across all sites reporting cases. While this dataset emphasizes feasibility for automated reporting, a minimum data standard will reduce risk of oversimplification of specifications.
  a. Additional data dictionary specifications as distinct data modules can be considered or added as population-wide PD registries mature. Such an approach addresses the problem of missing data when desired data elements are specified as “required if available” or optional. Sites with sufficient reporting capabilities, resources, or interest (e.g., neurology or movement disorder practices) may be incentivized to report on additional specified data elements.
10. Assess and ensure that recommended data elements are represented and mapped to standard concept codes. Gaps identified should be addressed with a strategy to develop, test, and create appropriate codes with appropriate standard development organizations.
  a. As an example, a consensus strategy to update the current ICD10 code for PD (G20) can be considered to separate out alternate diagnoses of a nonspecific parkinsonism or an uncertain early PD.
11. Support evaluation of data elements that are considered for population-wide PD registries, but will be more challenging to collect. Data elements can be proposed as provisional and tested before being approved within either a basic or higher tier data specification.
12. Support, develop, and incentivize a systematic and scalable validation process for population-based PD registries. As a starting point for discussion, the UCE-PD team has developed proof-of-principle processes and tools to support abstraction and case adjudication for PD registries.

*Registry implementation:*

13. Recommend that each state PD/parkinsonism registry maintain a standing scientific and patient advisory committee to ensure stakeholder engagement and alignment with consensus guidelines. A forum should be available where state PD/parkinsonism registries and the CDC NNCSS can communicate, share strategies, and align on national goals.
14. Prioritize automated reporting through certified EHR mechanisms. As eCR is now a mandatory component of the 2023 Medicare Promoting Interoperability incentive payment system (37), we recommend that an eCR specification be used as a preferred public health report system for PD registries.
15. Monitor and evaluate technologies and policies covering interoperability solutions as relevant to the development of a network of interoperable state and population-wide PD registries (Figure 2B). Examples include:
  a. Alignment with the United States Core Data for Interoperability (USCDI), the federally required set of data elements that certified EHR systems must support for interoperability. USCDI+ was recently announced as a possible domain-specific extension for which PD registries could be an ideal domain use case (38).
  b. Fast Healthcare Interoperability Resources (FHIR) standards hold promise for enabling interoperability between population-based registries and can support domain-specific data dictionaries (39).
  c. The Trusted Exchange Framework and Common Agreement (TEFCA) is a set of principles, technical requirements, and policies that support a nationwide system for securely sharing interoperable electronic health information (40). PD registries may be an ideal public health use case for the TEFCA network.

## Conclusion

With the advent of statewide PD registries, we believe now is the time to (re)address scope, design, implementation, validation, and interoperability issues. We call on PD registry owners and stakeholders to consider these gaps and recommendations as we work toward a feasible framework for a truly inclusive population-wide PD registry that serves as a trusted resource for public health, clinical care, and research.

## Data Availability

The raw data supporting the conclusions of this article will be made available by the authors, without undue reservation.
