# Supplementary material for: Parkinson’s disease population-wide registries in the United States: Current and future opportunities
Source: Front Digit Health. 2023 Mar 22;5:1149154. doi: 10.3389/fdgth.2023.1149154 (PMC10073707; doi:10.3389/fdgth.2023.1149154)
Supplement: Supplementary file 1 [file Datasheet1.docx]

Supplementary Material

Parkinson’s disease population-wide registries:
current and future opportunities

Allan D. Wu, MD^1,2,4*^, Andrew M. Wilson, MD^2,3^

^1^Division of Movement Disorders, Department of Neurology, Feinberg School of Medicine, Northwestern University, Chicago, IL.

^2^Department of Neurology, David Geffen School of Medicine, University of California Los Angeles (UCLA), Los Angeles, CA.

^3^Department of Neurology, Greater Los Angeles VA, Los Angeles, CA.

^4^Stanley Manne Children’s Research Institute, Ann & Robert H. Lurie Children’s Hospital, Chicago, IL.

*** Correspondence:**Allan D. Wu, MD
allan.wu@northwestern.edu

# Supplementary Material:

# S1. California Parkinson’s Disease Registry Specifications

CPDR v1.1 specifications represent initial proposed specifications in early 2018 when the mandatory requirement for reporting was announced.

The CPDR v3 specifications were the final requirements for mandatory reporting. No significant changes to the data element specifications were made in v3.1 which remain the current reporting specifications.

**Table S1: Comparison of CPDR specifications v1.1 (May 2018) and v3 (Aug 2018)**

| **Data Content Area** | **Requirement Optionality (CPDR v1.1)*** | **Requirement Optionality (CPDR v3)**** | **Field** |
| --- | --- | --- | --- |
| **Patient ID** | Required | Required | Name (Last, First, MI) |
|  | Required | Required | Date of Birth |
|  | Required | Required | Sex - (Gender) |
|  | Required if avail | Not present | Phone Number |
|  | Required if avail | Not present | Email Address |
|  | Required | Required | Patient Street Address (Street & No) |
|  | Required | Required | Patient Address City |
|  | Required | Required | Patient Address State |
|  | Required | Required | Patient Address Zip (Postal) Code |
|  | Required if avail | Optional | Social Security Number |
|  | Required | Required | Medical Record Number - MRN |
| **Patient Demographics** | Required | Required | Race |
|  | Required | Required | Ethnicity |
|  | Required if avail | Not present | Marital Status |
|  | Required if avail | Not present | Religion |
|  | Required if avail | Optional | Date Last Contact/Death |
| **Next of Kin** | Required if avail | Not present | Name (Last, First, MI) |
|  | Required if avail | Not present | Relationship |
|  | Required if avail | Not present | Address |
|  | Required if avail | Not present | Phone Number |
| **Patient Visit Information** | Required if avail | Optional | Attending Doctor |
|  | Required if avail | Not present | Referring Doctor |
|  | Required if avail | Optional | Consulting Doctor |
|  | Required if avail | Optional | Hospital Service |
|  | Required if avail | Not present | Date/Time Patient Arrived for Services |
|  | Required if avail | Not present | Date/Time Patient services ended |
|  | Required if avail | Optional | Admission Reason |
| **Physician Identifiers (Primary)** | Required | **Required** | Author NPI - Physician ID |
|  | Required | **Required** | Physician phone number |
| **Primary Diagnosis** | Required | **Required** | ICD-10/Diagnostic Term |
|  | Required | **Required (Optional if dx before 7/1/18)** | Month/Year of Diagnosis |
|  | Required | Not present | Comment |
| **Secondary Diagnosis** | Required if avail | Not present | Comorbid Condition(s) |
|  | Required if avail | Not present | Comment |
| **Disease Onset** | Required if avail | Optional | Onset Date, Onset of Symptoms |
|  | Required if avail | Not present | Comment |
| **Cardinal Signs / Symptoms of PD** | Required if avail | Not present | UPDRS: Mentation, Behavior, Mood |
|  | Required if avail | Not present | UPDRS: Activities of Daily Living |
|  | Required if avail | Not present | UPDRS: Motor Examination |
|  | Required if avail | Not present | UPDRS: Complications of Therapy |
|  | Required if avail | Not present | UPDRS: Hoehn and Yahr Staging |
|  | Required if avail | Not present | UPDRS: Schwab England Activities of Daily Living |
|  | Required if avail | Not present | UPDRS: Schwab England: with Dyskinesia |
|  | Required if avail | Not present | Clinical notes regarding general cardinal signs and/or symptoms of Parkinson's disease |
| **Surgical Treatments** | Required if avail | Not present | Deep Brain Stimulation, Neuroablative Procedures |
|  | Required if avail | Not present | Comments |
| **Medications** | Required if avail | Not present | Name |
| **(fields may repeat, Med 1, Med 2,…..)** | Required if avail | Not present | Identifier |
|  | Required if avail | Not present | Dose |
|  | Required if avail | Not present | Frequency |
|  | Required if avail | Not present | Comments |

*CPDR v1.1: California Department of Public Health. (May 2018). *The California Parkinson’s Disease Registry Implementation Guide version 1.1*.

**CPDR v3: California Department of Public Health. (Aug 2018). *The California Parkinson’s Disease Registry Implementation Guide Version 3.0*.

## S2. Data Elements Approved by EHR Vendor for Clinical Decision Support

In partnership with the EHR vendor, Epic Systems (Verona, WI) we selected several key review of symptoms (ROS) during usability testing with primary care and neurology clinicians. Once the key ROS elements were selected for a clinical decision support tool, we submitted the list of ROS elements to Epic Systems to build as standard interoperable discrete data elements. Once approved, these were then added as standard “Foundation” data elements to our local EHR and used to build the a PD ROS SmartForm tool. Data collected with this tool are now interoperable with any other Epic site that uses these data elements.

## Table S2. Smart Data Elements Approved by EHR Vendor for Interoperability

| **Epic SmartData Element (SDE) hierarchy / location** | **Data element** | **Status** |
| --- | --- | --- |
| DIAGNOSIS/PROBLEMS - NEUROLOGICAL - PARKINSON’S DISEASE | date of diagnosis | standard “date of diagnosis” was already available for conditions  (a PD-specific date of diagnosis element is still under review) |
|  | date of diagnosis uncertainty | approved for project |
| WORKFLOW - PD REGISTRIES - MOTOR | tremor  smaller handwriting  trouble with buttons or zippers  trouble with utensils  trouble turning over in bed  slowness of movement  muscle stiffness  loss of facial expression  voice softer | approved for project |
| WORKFLOW - PD REGISTRIES - GAIT/BALANCE | balance difficulty  walking difficulty  falls  shuffling gait  stooped posture  slow gait  difficulty standing from low chair  freezing of gait | approved for project |
| WORKFLOW - PD REGISTRIES - NON-MOTOR | loss of smell  fatigue | approved for project |
| WORKFLOW - PD REGISTRIES - PSYCHIATRIC | depression  anxiety  hallucinations  impulse control disorders | approved for project |
| WORKFLOW - PD REGISTRIES - AUTONOMIC | drooling  trouble swallowing  constipation | approved for project |
| WORKFLOW - PD REGISTRIES - SLEEP | restless leg syndrome  REM behavior disorder  insomnia | approved for project |
| WORKFLOW - PD REGISTRIES - COGNITIVE | memory difficulty  dementia | approved for project |

Epic Systems supports nearly 200,000 discrete data elements with slight variations in similar concepts. For example, there are 215 distinct tremor concepts, so what one medical group uses to code tremor may not be the same element chosen by another group. We worked with Epic to create a PD REGISTRIES section where concepts that are reportable to PD registries in the future can be vetted and kept uniform across all Epic users. This makes it easier to find appropriate data elements future automated aggregation of similar data being exported from Epic sites.

**S3. UCE-PD Classification Proposal**

Table S3 provides a framework summary and conceptual definitions of each classification.

The availability of “info” (as reflected in the “with info” and “missing info”), refers to the availability of documentation such that, if there was the opportunity to document features that would support Probable PD if they were present, then one would anticipate that those would have been documented. In most typical cases, the “with info” feature is met when there is availability of neurology evaluations (“neuro evaluations”). Probable PD does not have a “missing info” subcategory, as it is a prerequisite to have supporting documentation to be assigned to that level. The “Not PD (missing-info)” is the only category with no detection of any PD/parkinsonism that would qualify for reporting to a PD/parkinsonism registry. This category will account for the vast majority of all patients contained within a health system and is included for completeness. In practice, there will be cases reported to a PD/parkinsonism registry (yes for that feature) but turn out to have active documentation that refutes the diagnosis of PD. These qualify for the “Not PD (with info)” category and are of practical interest to registries to detect. For purposes of high-level classification, both Not PD versions can be combined into one overview category of Not PD.

Concept definitions are similar to clinical definitions used in practice. Concept definitions are used routinely by clinicians to assess the likelihood of a given diagnosis or not. Does the patient “fit” this concept of a clinical condition of Parkinson’s disease or not? These concept definitions are not operationalized in this document and have not yet been assessed for formal phenotyping definitions. We intend that these proposed concept definitions may guide further development, implementation, and testing of formal phenotyping efforts.

**Table S3. UCE-PD Diagnostic Classification Framework**

| *Classifications* | *Principles* | *Conceptual definition* |
| --- | --- | --- |
| Probable PD | documentation actively supports PD | Records available have documented parkinsonism (bradykinesia with at least 1 of rigidity or rest tremor) ANDhas a combination of neurologist evaluation(s), dopamine transporter scan (DaTscan) with dopaminergic deficit, dopaminergic-medication responsiveness, enough time or repeated evaluations, and relative lack of red flags against PD to support a typical PD diagnosis(AND does not fulfill any Possible PD categories) |
| Possible PD  – missing info | *n/a* | Records available for review raises the possibility of a PD diagnosis, but the chart does not have enough relevant documentation to classify in Probable PD, Possible PD w/info or Not PD categories.Typically, neurology evaluations are not available (i.e. there is no documentation of an opportunity to record relevant symptoms or signs if they were present or absent). |
| Possible PD– with info | documentation does not actively or consistently support or refute PD | Records available for review contain documentation that includes the possibility of a PD or a neurodegenerative parkinsonism syndrome.The documented information is not sufficient to confidently classify in either Probable PD or Not PD.Typically, neurology evaluations are available (i.e. there is documented opportunity to record relevant symptoms and signs and is not sufficient to classify as Probable PD or Not PD). |
| *Possible PD* *– too early subclassification* | *Support for PD but not sufficient for Probable PD;* *typically 0-5 years of date of diagnosis* | *Documented symptoms or signs are not sufficient to reach Probable PD status.**Typically neurology evaluations exist such that the opportunity would be considered present to document findings sufficient for Probable PD.**Typically this category is used within 0-5 years of symptom onset.* |
| *Possible PD – neurodegenerative-parkinsonism in ddx subclassification* | *diagnosed with neurodegenerative parkinsonism syndrome* *OR  considered for neurodegenerative parkinsonism* | *Documentation in support of diagnosis should emphasize most contemporary evaluations and neurology evaluations.**Documentation in support of a parkinsonism syndrome diagnosis is either:**sufficient to support (favor the diagnosis of) any of the neurodegenerative parkinsonism syndromes;**OR**sufficient to consider (as an alternative or within differential diagnosis) any of the neurodegenerative parkinsonism syndromes****Neurodegenerative parkinsonism syndromes include: PD, PSP, CBD, MSA, SND, LBD or other atypical parkinsonisms* |
| *Possible PD* *– too complex subclassification* | *considered for non-neurodegenerative parkinsonism syndrome(s)* *OR**have conditions that confound ability to document features for Probable PD* *OR**conflicting or cannot-decide upon a diagnosis with multiple neuro evals* | *Documentation in support of diagnosis should emphasize most contemporary evaluations and neurology evaluations.**Documentation available supports any of below:**diagnosis considerations include non-neurodegenerative parkinsonism syndromes (vascular parkinsonism, drug-induced parkinsonism, normal pressure hydrocephalus);* *OR**presence of conditions that would limit the ability to document findings sufficient for Probable PD (i.e. if the conditions were not present and if Probable PD findings were present, they would have been likely documented); e.g. severe homebound dementia, traumatic brain injuries, multiple large strokes, cerebral palsy, spinal cord injuries, assessments during acute hospitalization;*  *OR**inconsistent diagnosis for a parkinsonism syndrome. Typically, there are multiple contemporary neurology evaluations, yet either neurologists disagree; or neurologists assess and cannot decide on a diagnosis (and at least one alternate dx is non-neurodegenerative or confounded - otherwise would capture solely in “neurodegenerative-ddx”)* |
| Not PD (with info) | documentation actively refutes PD | Records available contains documented evidence to refute neurodegenerative parkinsonism or a PD diagnosis.Typically, has documented neurologist evaluations who would have observed parkinsonism if present and documented that relevant signs and symptoms were not present. |
| Not PD (missing info) | *n/a* | Records available for review does not have any information to raise a possibility of a PD diagnosis. |

# *Abbreviations:*

# *PD=Parkinson’s disease; PSP=progressive supranuclear palsy; CBD=corticobasal degeneration; MSA=multiple systems atrophy; SND=striatonigral degeneration; LBD=Lewy body dementia*
